# Cocktails of defined chemical compounds: sufficient to induce totipotency in embryonic stem cells

**DOI:** 10.1038/s41392-022-01184-8

**Published:** 2022-09-19

**Authors:** Wilfried A. Kues, Dharmendra Kumar

**Affiliations:** 1 Friedrich-Loeffler-Institut, Federal Research Institute for Animal Health, Biotechnology, Stem Cell Physiology, 31535 Neustadt, Germany; 2 Animal Physiology and Reproduction Division, ICAR-Central Institute for Research on Buffaloes, Hisar, Haryana, 125001 India

**Keywords:** Embryonic stem cells, Pluripotency, Stem-cell biotechnology, Target identification

In a recent work published in *Nature*, Hu et al.^1^ report that a cocktail of three small chemicals is sufficient to induce totipotency in murine embryonic stem (ES) cells. Earlier this year, two other groups identified different mixtures of chemicals, which also seem to do this job,^2,3^ taken together this opens a new approach to capture totipotency in vitro, and to understand the underlying pathways and their interaction.

**Fig. 1:**
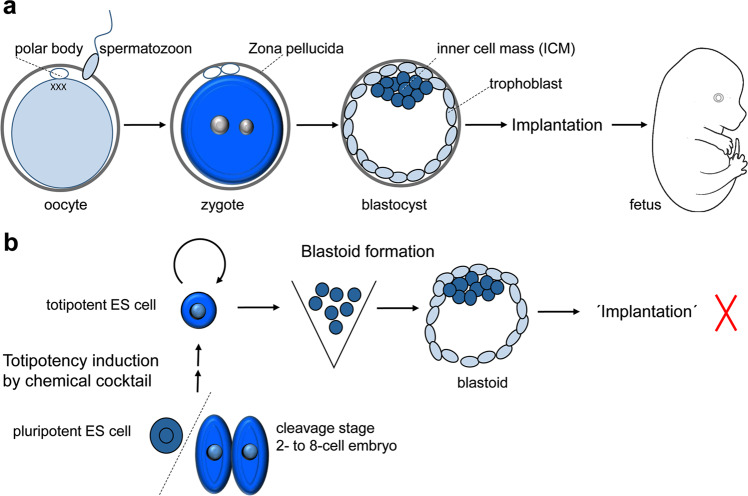
In vivo and in vitro totipotency. **a** Totipotency during ontogenesis: fertilization sparks cellular reprogramming resulting in a totipotent zygote, which can develop into a fetus after implantation. In vivo totipotency is a rapidly passing condition present only in zygote and blastomeres of the first cleavage division. **b** In vitro totipotency: Totipotency can be induced and captured in pluripotent ES cells or early cleavage stages by cocktails of defined chemical compounds. By a 3D culture environment, the totipotent stem cells can be triggered to self-organize into a blastocyst-like structure—a blastoid—which is able to initiate implantation. However, no further structured development has been achieved to date indicating that something is still missing

During murine ontogenesis, cellular totipotency is achieved in the zygote and two-cell cleavage stage (Fig. 1). ES cells, which are derived from blastocyst stage, are pluripotent and they can develop into all cells of the adult body, but not to extraembryonic cells. Hu and coworker employed ES cell lines carrying a fluorescent reporter construct driven by a MERVL (murine endogenous retrovirus L) promoter to screen a chemical library of ~3000 compounds. MERVL is an endogenous retrovirus, which is exclusively expressed in two-cell embryos. The chemicals which could induce MERVL-tdTomato expression were then assessed in a combinatorial approach, finally defining a cocktail of three compounds of TTNPB, 1-azakenpaullone, and WS6,^1^ which resulted in a constant upregulation of the totipotency reporter.

The resulting chemically induced totipotent stem cells (ciTotiSCs) were able to maintain long-term totipotency in in vitro culture with a stable karyotype, expressed totipotency marker genes such as Zscan4, Zfp352, Tcstv1, Tcstv3, and Sp110, and exhibited downregulation of pluripotency marker genes such as Oct4, Nanog, Sox2, Tdgf1, and Zfp42. Upregulation of totipotent genes and downregulation of pluripotent genes were confirmed with bulk and single-cell RNA-sequencing. The transcriptome analyses and genome-wide chromatin accessibility of ciTotiSCs evaluated by transposase-accessible chromatin sequencing support the close resemblance of ciTotiSCs with mouse two-cell blastomere. DNA methylation analysis was performed, and the ciTotiSCs showed a hypomethylated genomic architect similar to zygotes or two-cell mouse embryos.^1^ Single-cell transcriptome analysis of the ciTotiSCs suggests that they are closer to the two-cell stage than the previously described totipotent blastomere-like cells (TBLCs).^4^

In addition to transcriptome and epigenome analyses, metabolome evaluation revealed that ciTotiSCs exhibited metabolic features similar to totipotent cells. The ciTotiSCs were able to differentiate into embryoid bodies in vitro and formed teratomas, and formed both embryonic and extraembryonic lineages after aggregation with host embryos. For a stringent evaluation of totipotency, reporter-labeled (with ubiquitous expression) ciTotiSCs were aggregated with mouse eight-cell stage embryos and transferred to pseudo-pregnant female mice, and chimerism was evaluated in E7.5 conceptuses. The reporter-labeled cells integrated into epiblast, extraembryonic ectoderm, and ectoplacental cone, and in the later stage of embryonic development (E13.5) these cells contributed to the formation of parts of the placenta and entire yolk sac.^1^ These results were verified with single-cell RNA-sequencing, which also revealed that ciTotiSCs contributed to the formation of extraembryonic trophoblast and yolk sac cell types, and confirmed the exclusive expression of lineage-specific marker such as visceral yolk sac cells (Apoa4, Fxyd2, Entpd2), spongiotrophoblast cells (Tpbpa, Rhox9) and syncytiotrophoblast cells (Itm2a).^1^ Strikingly, ciTotiSCs are capable to produce germline chimeras after aggregation with eight-cell embryos.

Recently, two other groups showed the induction of totipotency by different cocktails of small chemicals. As Hu et al., they used MERVL reporter constructs for screening,^2,3^ Xu et al. also used a Zscan4-Emerald reporter.^2^ Xu et al. achieved totipotent potential stem (TPS) cells from mouse extended pluripotent stem cells and 2-cell mouse embryos by screening a chemical library and defining a cocktail of compounds, which includes CD1530, VPA, EPZ004777, and CHIR 99021.^2^ The molecules CD1530, VPA, EPZ004777 regulate totipotency synergistically. Yang et al.^3^ reported that the remodeling of pericentromeric region of heterochromatin and H3K4me3 domains reprograms mouse ES cells to totipotent-like stem cells (TLSCs). For the remodeling of chromatin, a combination of small molecules such as SGC0946 (a potent, selective inhibitor of DOT1L (disruptor of telomeric silencing 1-like, a histone H3K79 methyltransferase)) and AS8351 (an inhibitor of lysine demethylase 5B) was used. These molecules increased the expression of MERVL and totipotency-associated genes in mouse pluripotent stem cells and promotes the transition from the pluripotent to TLSCs. Both TPSs and TLSCs were able to self-organize to form blastoids, which had the potential to implant and initiate decidualization, however without the formation of a fetus.

Among these molecules, CD1530 is an agonist of retinoic acid receptor γ (RARγ), VPA is an inhibitor of histone deacetylase (HDAC), and EPZ004777 and SCG0946 are DOT1L inhibitors, whereas CHIR 99021 play an important role in activating wingless and Int-1 (Wnt) signaling by inhibiting glycogen synthase kinase 3β (GSK-3β) signaling pathways. AS8351 supports the transition to totipotency via inhibition of histone demethylase. One road of totipotency induction occurs due to inhibition of DOT1L and HDAC, and activation of RARγ signaling. TTNPB is an analog of retinoic acid (RA) that selectively activates RAR and improves the reprogramming efficiency of the cells. 1-Azakenpaullone is a selective inhibitor of GSK-3β, which regulates multiple signal transduction pathways, and is also a key component of the network responsible for maintaining stem cell pluripotency, and WS6 is an inhibitor of Erb3 binding protein 1 and the NFκB kinase pathway. CHIR 99021 is valuable for promoting the proliferation of totipotent stem cells and essential for maintaining the in vivo developmental potency of these cells. Another road to totipotency seems to be the spliceosome inhibition via pladienolide B.^4^ Taken together these publications demonstrated the induction of totipotency using different defined chemical compounds with partially overlapping effects on the pathways of Wnt signaling, RA signaling, histone methylation and deacetylation, and spliceosome inhibition. The identified molecular pathways will stimulate further research to optimize the protocol for induction and maintenance of totipotency.

The ability of totipotent cells to generate artificial fetuses with full developmental capacity remains to be assessed (Fig. 1). The currently available totipotent cells seem to be competent for artificial blastocyst development and initiation of implantation, however unable for ordered gastrulation and embryo formation without support from a host embryo. One milestone toward the formation of a fully artificial embryo was the recent demonstration that the imprinting barrier can be overcome by the generation of live mouse offspring from unfertilized oocytes through targeted DNA methylation rewriting of seven imprinting control regions using CRISPR activation and interference.^5^
